# No Evidence of Viral Transmission following Long-Term Implantation of Agarose Encapsulated Porcine Islets in Diabetic Dogs

**DOI:** 10.1155/2014/727483

**Published:** 2014-06-05

**Authors:** Lawrence S. Gazda, Horatiu V. Vinerean, Melissa A. Laramore, Richard D. Hall, Joseph W. Carraway, Barry H. Smith

**Affiliations:** ^1^The Rogosin Institute-Xenia Division, 740 Birch Road, Xenia, OH 45385, USA; ^2^The Rogosin Institute, New York, NY 10021, USA; ^3^Florida International University, Miami, FL 33199, USA; ^4^Bob Evans Farms, Inc., New Albany, OH 43054, USA; ^5^NAMSA, Northwood, OH 43619, USA; ^6^NewYork-Presbyterian Hospital, Weill Medical College of Cornell University, New York, NY 10021, USA

## Abstract

We have previously described the use of a double coated agarose-agarose porcine islet macrobead for the treatment of type I diabetes mellitus. In the current study, the long-term viral safety of macrobead implantation into pancreatectomized diabetic dogs treated with pravastatin (*n* = 3) was assessed while 2 dogs served as nonimplanted controls. A more gradual return to preimplant insulin requirements occurred after a 2nd implant procedure (days 148, 189, and >652) when compared to a first macrobead implantation (days 9, 21, and 21) in all macrobead implanted animals. In all three implanted dogs, porcine C-peptide was detected in the blood for at least 10 days following the first implant and for at least 26 days following the second implant. C-peptide was also present in the peritoneal fluid of all three implanted dogs at 6 months after 2nd implant and in 2 of 3 dogs at necropsy. Prescreening results of islet macrobeads and culture media prior to transplantation were negative for 13 viruses. No evidence of PERV or other viral transmission was found throughout the study. This study demonstrates that the long-term (2.4 years) implantation of agarose-agarose encapsulated porcine islets is a safe procedure in a large animal model of type I diabetes mellitus.

## 1. Introduction

In Type I diabetes, the insulin-producing islets of Langerhans, scattered throughout the pancreas, are selectively destroyed by an apparent autoimmune process. Daily and often multiple injections of exogenous human recombinant insulin are the current standard of care. Despite continual advances in insulin therapy and the fact that some patients have now survived for more than 50 years with insulin therapy, the quality of life can be difficult owing to the nonphysiological delivery of an insulin bolus. The transplantation of the entire human pancreas from a deceased organ donor provides another therapeutic option for diabetic patients, especially, for those patients already receiving immunosuppressive therapy for a kidney allograft [1]. Alternatively, the insulin-producing islets can be isolated from the majority of the pancreas and transplanted alone as free islets [2]. As noted above, a necessary component of the transplantation of an allogeneic pancreas or the islets alone is the need for lifelong immunosuppressive therapy. Because complications from such immunosuppressive therapy can include increased susceptibility to infections, malignancy, neurotoxicity, and nephrotoxicity, allotransplantation must be carefully considered and is often not suitable for young patients [3]. Further, the availability of human donor pancreas is extremely limited with only about 2,000 pancreas donors per year [4]. Worse, only about 16% of procured pancreases meet the criteria for transplantation and less than 3% are used for islet isolation [4, 5].

To overcome the major hurdles of organ availability and immunosuppression, we have reported the ability of porcine islets, encapsulated in an agarose-agarose macrobead, to restore normoglycemia in diabetic animal models [6]. The use of pigs as an islet source provides an unlimited number of organs for islet isolation and encapsulation. Further, porcine insulin is nearly identical to human insulin, differing in only a single amino acid and it has been used in the clinic since its discovery in 1922 [7]. The double layered agarose encapsulation of the porcine islets also provides at least some protection from recipient immune responses to the xenogeneic islets and is likely to ameliorate the need for immunosuppressive therapy. In fact, we have previously shown the porcine islet macrobeads to enable the discontinuation of exogenous insulin for more than 6 months (study termination) in spontaneously diabetic BB rats without immunosuppressive therapy [6].

The current study was initiated to further investigate the effects of multiple implantations of encapsulated porcine islets to pancreatectomized diabetic dogs (*n* = 3 dogs that received islet macrobeads and *n* = 2 dogs that received exogenous insulin only). Because human diabetic patients implanted with macrobeads would be chronically exposed to porcine islets, we further set out to assess the viral safety of the macrobeads before implantation and also screened various canine tissues for evidence of viral transmission after more than 2 years of porcine islet macrobead exposure.

## 2. Materials and Methods

### 2.1. Animals

A total of six male devocalized Beagle dogs (Ridgelan Farms, Inc., Mount Horeb, WI) were received at approximately 20 weeks of age. The dogs were individually housed in stainless steel cages, 32′′ W × 42′′ L × 32′′ H, with rubber-coated mesh flooring and a stainless steel platform, 32′′ × 40′′. Science Diet Growth (Hill's, 6730) was provided twice daily and clean municipal water provided* ad libitum*. Viokase (Henry Schein, 9758341) powder was added to food at a dose of 1 tsp/meal following pancreatectomy. Room temperature was maintained at 18–28°C with relative humidity of 30–70%. A 12-hour light cycle was maintained throughout the study with lights on at 0700 hours. All study protocol procedures were approved by The Rogosin Institute Animal Care and Use Committee (IACUC). The Rogosin Institute-Xenia Division animal facility holds Full Accreditation status awarded by the Association for Assessment and Accreditation of Laboratory Animal Care, International (AAALAC, Int.).

### 2.2. Isolation of Porcine Islets and Production of Porcine Islet Macrobeads

Donor islets were prepared from Newsham sows over two years of age and with multiple parities. After electrical stun and exsanguination (Bob Evans Farms, Xenia, OH), pancreata were retrieved and transported to the islet isolation laboratory in cold Hank's balanced salt solution (HBSS; Mediatech, 21-020) on ice. Warm ischemia times averaged 12.26 minutes while cold ischemia times ranged from 30 minutes to one hour. Islet isolation was carried out as previously described [6]. Briefly, after trimming the gland of fat and connective tissue, the main pancreatic duct was cannulated and injected with HBSS containing 2.0 g/L collagenase P (Roche, 11213873001) and 0.01 g/L DNase (Sigma Aldrich, D5025). Twice the gram weight of the pancreas (in mL) was perfused through the pancreatic duct at a rate of 50 mL/min at 18°C. Islets were purified on discontinuous Eurocollins (Mediatech, Inc., 99-408)-Ficoll (Sigma Aldrich, F9378) gradients of densities 1.105 g/cm^3^, 1.095 g/cm^3^, and 1.055 g/cm^3^ in 50 mL polystyrene conical tubes. The tubes were centrifuged at 2000 RPM, and islet-containing layers were manually collected. 500 islet equivalents were encapsulated in each agarose-agarose macrobead as previously described [6]. Islet macrobeads were maintained in RPMI containing 2.5% porcine serum (Mediatech, 35-041) and 1% antibiotic/antimycotic (A/A; Gibco Life Technologies, 15240) in an atmosphere of 3–5% CO_2_ and air at 36–38°C. Macrobeads were examined for uniformity, collected the day prior to implant, and were aliquoted to 175 mL conical tubes. A maximum of 400 macrobeads per tube were stored overnight at room temperature (18–28°C) in RPMI (Gibco Life Technologies, 22400) containing 1% A/A.

### 2.3. Microbiological and Viral Screening

Prior to islet macrobead implantation, representative samples of macrobeads and culture media were sent to MicroTest Laboratories, Inc. (Agawam, MA) for product bioburden testing (ANSI/AAMI/ISO 11737-1 Part 1); see Table 1. Macrobeads were aseptically crushed and extracted with Fluid D (EMD Millipore, 1.46483.0006) while the culture media was tested as supplied. Using USP 〈61〉 membrane filtration, each sample was filtered onto a 0.45 *μ*m membrane (EMD Millipore, HVLP), which was then transferred to tryptic soy agar (TSA) plates and cultured at 32.5°C ± 2.5°C for 3–5 days (aerobic, anaerobic, and aerobic spore formers). Additional samples were filtered onto 0.45 *μ*m membranes, which were transferred to Sabouraud dextrose agar (SDA) plates and incubated at 22.5°C ± 2.5°C for 5–7 days (yeast and mold). A USP 〈62〉 microbial limits test for* Salmonella sp.* was also performed by incubation of samples in lactose broth for 48 hours at 30–35°C, aliquoted 1 mL/tube to SC/TT tubes, followed by an additional 24 hours at 32.5°C ± 2.5°C. Upon completion of incubation, contents were streaked to brilliant green agar (BGA), xylose lysine desoxycholate (XLD) agar, and bismuth sulfite (BS) agar plates for 48 hours at 32.5°C ± 2.5°C. All bioburden testing included negative media controls and appropriate positive media controls (*Escherichia coli* [ATCC #11229], aerobic;* Clostridium sporogenes* [ATCC #11437], anaerobic;* Bacillus subtilis* [ATCC #6633], aerobic sporeformer;* Candida albicans* [ATCC #10231] yeasts and molds). Colonies were counted using a Reichert Quebec darkfield colony counter. For each class of organism, the total recoverable bioburden as CFU/SIP was calculated (culture media SIP = 5 mL per category; macrobead SIP = 1 bead).

Culture media and islet macrobeads were also sent to Iowa State University, Veterinary Diagnostic Laboratory (VDL; Ames, IA) for additional microbiological testing including porcine virology prior to macrobead implantation, as summarized in Table 1. Porcine virology tests included: porcine reproductive and respiratory syndrome virus (PRRSV; RT-PCR using Qiagen, 282315), swine influenza virus (SIV; RT-PCR using Qiagen, 282615), porcine endogenous retrovirus (PERV; RT-PCR) [8], porcine enterovirus (PEV; RT-PCR) [9], porcine respiratory corona virus (PRCV; RT-PCR using Qiagen, 283615), transmissible gastroenteritis virus (TGEV; RT-PCR using Qiagen, 283615), porcine circovirus (PCV; PCR) [10], porcine lymphotropic herpes virus type-1 (PLHV-1; PCR) [11], swine hemagglutinating encephalomyelitis virus (sHEV; RT-PCR) [12], porcine parvovirus (PPV; PCR) [13], porcine cytomegalovirus (PCMV; PCR) [14],* Mycoplasma hyopneumoniae* (PCR) [15], pseudorabies virus (PRV; virus isolation), encephalomyocarditis virus (EMCV; virus isolation), group A rotavirus (antigen capture ELISA), and* Chlamydia* spp. (antigen capture ELISA). Each RT-PCR/PCR assay included a positive control, a no template control, and was performed according to standard operating procedures of VDL.

At the time of the surgical procedure for the second macrobead implant (day 215 post-1st implant), or sham surgery for non-implant controls, white blood cells and biopsies of mesenteric lymph node and tonsil were collected from all study animals and immediately snap-frozen. Samples were shipped on dry-ice to BioReliance (Glasgow, Scotland) for assessment of PERV by quantitative real-time PCR (qRT-PCR). The appropriate controls including: positive controls (negative control nucleic acid spiked with viral target sequence), post-extraction spike controls (to assess target specific PCR inhibition), exogenous internal positive controls (to establish that all negative PCR results were truly negative and not due to failed amplification), no-template controls (to monitor aerosol and reagent contamination), negative controls (to monitor reagent contamination), and sentinel extraction controls (to assess possible airborne sample to sample cross-contamination) were conducted in triplicate with each assay.

At necropsy, samples of retrieved islet macrobeads and swabs of the peritoneal cavity were sent for Sterility testing per USP 〈71〉 via membrane filtration and product immersion to MicroTest Laboratories, Inc. Using a direct cultivation and cell culture method, retrieved islet macrobeads were screened for the presence of Mycoplasma (MicroTest Laboratories, Inc., per the FDA Points-To-Consider in the Characterization of Cell Lines used to produce Biologicals). Macrobeads were also screened for the presence of Bacterial Endotoxin by MicroTest Laboratories, Inc. following the procedure depicted in the FDA 1987 Guidance for Bacterial Endotoxin Testing.

Samples of retrieved macrobeads, white blood cells, bone marrow, mesenteric lymph node, small intestine, liver, kidney, heart, lung and central nervous system (brain) were collected aseptically, snap-frozen, and sent to BioReliance for viral screening. The six different viruses screened for in necropsy tissues are shown in Table 2. Virus selection was based on their ubiquitous nature in swine (sEMCV, PLHV, sHepE, PCV, PCMV, and PERV), or their specific presence in source animals (PCV, PERV), or their trans-species infectivity potential (sEMCV, PLHV, sHepE, PCMV, and PERV), and clinical significance (all). Samples were assessed by qRT-PCR for the presence of specific porcine nucleic acid sequences from both control dogs and islet macrobead implanted dogs. Probe sequences used in the viral screening process are proprietary but are available upon specific request. Testing was conducted in accordance with the Principles of Good Laboratory Practice and under the regulations described in the United States Federal Register 21 CFR Part 58.

### 2.4. Surgical Procedures

Animals were sedated with acepromazine at a dose of 0.05 mg/kg IM (Henry Schein, 356-7290) and anesthetized with ketamine at a dose of 5 mg/kg (Fort Dodge Animal Health, 9952949), diazepam at a dose of 1 mg/kg (Butler, GNR04054) and isoflurane gas at 1–3% (Henry Schein, 982-2413). During surgery all animals received buprenorphine (Butler, GNR02300) at doses of 0.005–0.020 mg/kg IM during surgery and every 8–12 hours after surgery, continuing for 2-3 days; and cefotaxime (claforan antibiotic; Henry Schein, 852-1633) at a dose of 20 mg/kg IV during and 8 hours after surgery. At implant, the abdominal, subcutaneous, and cutaneous incisions were closed with 3-0 vicryl (Ethicon, J333), 3-0 vicryl, and 3-0 nylon (Ethicon, J669) sutures and 6.70 mm × 3.90 mm precise skin staples (3M Health Care), respectively. At pancreatectomy, the abdominal, subcutaneous, and cutaneous incisions were closed with 2-0 vicryl (Ethicon, J332), 4-0 polymend (Veterinary Products Laboratories, V-397-1), and 3-0 nylon (Ethicon, J669H) sutures, respectively.

Fourteen weeks following arrival, all animals underwent pancreatectomy for the induction of insulin-dependent diabetes. The pancreas was exposed by a peritoneal midline incision and removed with blunt dissection. Following induction, animals were maintained on Humulin 70/30 insulin (Eli Lilly, 029363) at doses to attempt morning (0900 hr) and evening (1800 hr) normoglycemia. At the time of pancreatectomy, the majority of the omentum was also removed.

### 2.5. Porcine Islet Macrobead Implantations

On day 81 after pancreatectomy, three animals received a 1st implant of porcine islet macrobeads at a dose equivalent to 4× daily insulin requirements. The three dogs that received the first implant underwent a second implant (dose = 3×) of islet macrobeads 215 days after the 1st implant. Insulin requirements of recipients and the corresponding number of porcine islet macrobeads received are given in Table 3. At the time of the second implant, nonimplanted control animals received a sham surgery. Animals receiving porcine islet macrobeads were started on pravastatin (80 mg/day; Teva Pharmaceuticals) 48 hr prior to a 1st implant and continued daily throughout the course of study.

Because the* in vivo* environment of the dog abdomen would likely impair insulin production from implanted macrobeads as compared to the carefully controlled* in vitro* culture environment, we opted to implant dogs with enough macrobeads to provide 4 times (4× dose for the 1st implant) or 3× (3× dose for the 2nd implant) the amount of each dog's daily exogenous insulin requirement. Insulin production during a 24 hr period of* in vitro* culture was determined once a week prior to macrobead implantation for each batch of islet macrobeads (see below) so that an average quantity of insulin secretion per macrobead in the four weeks prior to each implant could be calculated (see Table 3). The daily exogenous insulin requirements of each macrobead implanted dog were multiplied by 4 (first implant) or 3 (second implant) and enough macrobeads implanted to provide either a 4× or 3× dose of insulin. As insulin production varies with every batch of islet macrobeads, each preparation of islet macrobeads was evenly divided amongst the different recipient dogs such that each animal received a proportion of each batch of macrobeads based on exogenous insulin requirements. For the first implant, each dog received 1118–1380 porcine islet macrobeads with a total macrobead weight of 0.31–0.36 kg. For the second macrobead implant each dog received 1096–1904 porcine islet macrobeads weighing 0.29–0.49 kg. Average macrobead age (time after isolation and encapsulation) was 13.8 weeks for all dogs at the time of the first implant and 14.1 weeks for the second implant.

A peritoneal midline incision was made and the site of pancreatectomy was examined for the presence of any residual pancreatic tissue just prior to placing the macrobeads into the peritoneal cavity. Immediately prior to implant, macrobeads were washed three times with RPMI containing 1% A/A and were gently placed into the peritoneal cavity by use of a sterile plastic spoon. As needed, animals were manually fed Hill's Prescription Diet A/D (5670), Hill's canned Science Diet Growth (6680), and/or Nutri-Cal (Evsco Pharmaceuticals, 01311) to maintain normoglycemia during the first 24–48 hours after implant.

### 2.6. Clinical Observations

Individual animal medical observations were recorded daily throughout the study. Observations included appetite, bowel movements, body weight, blood glucose (Accu-Chek Simplicity BG monitor and Chemstrips; maximum value of 600 mg/dL: Roche Diagnostics), urine glucose, and insulin therapy.

### 2.7. Intraperitoneal Sample Collections

Intraperitoneal fluid was collected during a glucose/arginine challenge procedure and samples tested for the presence of porcine C-peptide as follows. Following light anesthesia, an IV catheter was inserted into the peritoneal cavity and 10 mL/kg of a 16.7 mM glucose and 5 mM arginine solution were injected. This infusion procedure was carried out for two of the implanted dogs (FOX-1 and KOX-1). HSX-1 received the glucose/arginine challenge, though intraperitoneal fluid was only collected from the initial 30–35 minute timepoint following challenge.

### 2.8. Porcine Insulin and C-Peptide Assays

Standard radioimmunoassays from Linco Research, Inc. were used for the detection of porcine insulin (PI-12 K, sensitivity of 2 *μ*U/mL) and porcine C-peptide (PCP-22 K, sensitivity of 0.1 ng/mL). Assays were run according to the manufacturer's instructions with samples in duplicate and reference, standards, and controls in triplicate.

### 2.9. Necropsy

Complete necropsies were performed on day 867 after 1st implant of porcine islet macrobeads. Following anesthesia, the collection of blood, and exsanguination, macrobeads were randomly retrieved for virology (*n* = 60; snap frozen, 6 macrobeads/tube), histopathology (*n* = 50; fixed in 10% neutral buffered formalin (NBF), 5 macrobeads/tube) or microbiology (*n* = 12; snap frozen, 6 macrobeads/tube). Macroscopic observations were noted, and major organ systems and tissues photographed. Weights of major organs were recorded. Tissue samples, including porcine islet macrobeads, were sent to BioReliance for viral screening or sent to Pathology Associates International (PAI, A Charles River Company; West Chester, OH) for histopathology.

### 2.10. Histopathology

At the time of pancreatectomy, both pancreas and omentum were fixed in 10% NBF for 24 hrs, washed, and stored in 70% ethanol. At necropsy, the following tissues were collected, fixed as above, and sent to PAI: heart, spleen, liver, kidneys, brain, testes, duodenum, jejunum, ileum, mesentery, adrenal glands, stomach, lungs, diaphragm, abdominal musculature, bone (sternum), spinal cord, sciatic nerve, epididymus, eyes, submandibular lymph nodes, bladder, muscle (thigh), thymus, thyroid, and parotid salivary gland. Tissues were embedded in paraffin, and 5 *μ*m sections were stained with haematoxylin and eosin (H&E). Samples were analyzed by a Diplomat of the American College of Veterinary Pathology, and the macroscopic and histopathological findings were documented. All histopathology was performed according to standard operating procedures of PAI.

## 3. Results

### 3.1. Preimplant Macrobead Screening

Culture media and macrobead samples were found to be negative for bacterial growth and* Salmonella sp.* throughout the observation periods. Prior to the first macrobead implant, 6 macrobead preparations and samples of the 6 corresponding culture medias were screened for a panel of porcine viruses and microbial agents as described above and shown in Table 1. All macrobead and media samples tested positive by RT-PCR for PERV. All other macrobead and media samples were negative for the screened viruses.

Prior to the second macrobead implant, 2 randomly selected macrobead preparations and samples of the 2 corresponding culture media were screened for the same panel of porcine viruses and microbial agents as the first implant macrobeads. Macrobead and media samples were positive for PERV and negative for all other screened agents.

### 3.2. Insulin Requirements and Individual Blood Glucose

**Figure 1:**
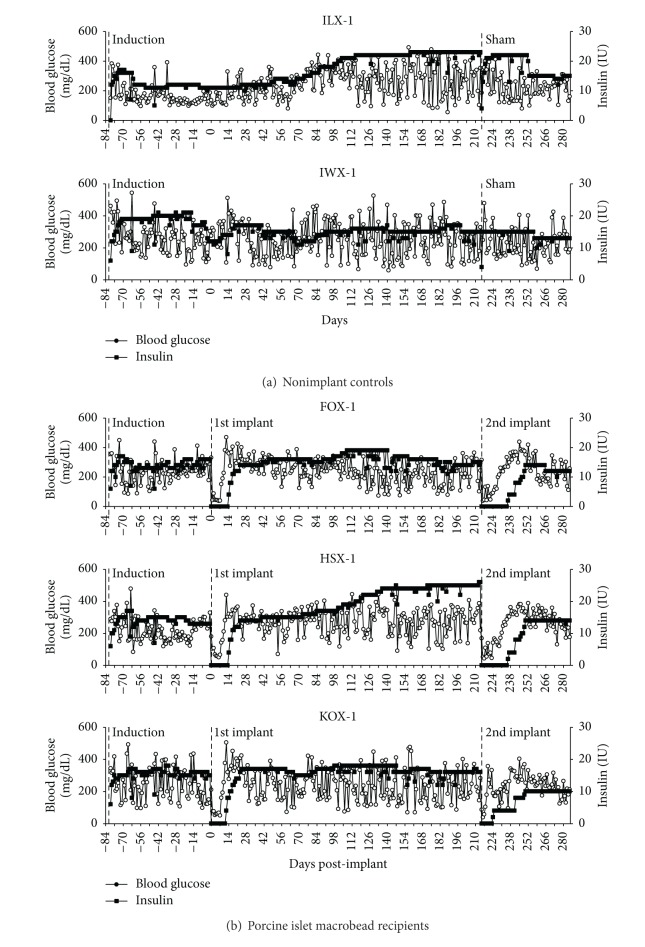
Blood glucose levels of study animals are shown as an average of daily AM and PM blood glucose levels (open circles). Exogenous insulin totals are graphed as a sum of daily AM and PM insulin units received (closed squares). Control animals (did not receive porcine islet macrobeads) are shown in (a) ILX-1 and IWX-1. Animals that received porcine islet macrobeads are shown in (b) FOX-1, HSX-1, and KOX-1.

Following pancreatectomy, insulin requirements were adjusted for each dog over a 4-week period. The dogs were considered to have exogenous insulin requirements established by week 4after induction. Mean daily insulin requirements between weeks 4-5 ranged from 11 to 20 IU per dog (Figure 1).

The three implanted dogs remained insulin-free for 12–14 days after the first implant of islet macrobeads, at which time the animals were started on 4 units of insulin in the morning and 4 units in the evening in response to rising blood glucose levels (Figure 1(b)). Exogenous insulin was gradually increased over the next 1-2 weeks until blood glucose levels stabilized. Preimplant insulin requirements were reestablished by 3 weeks following transplantation. The 2 control dogs did not undergo macrobead implantation or sham surgery at the time of the first implant.

Following the second islet macrobead implant, the three implanted dogs began exogenous insulin therapy on day 9 (KOX-1) or 21 (FOX-1 and HSX-1). Presecond implant insulin requirements were reestablished for the dogs after the 2nd implant on day 148 (FOX-1), day 189 (KOX-1), and >day 652 (HSX-1) after 2nd implant.

### 3.3. Serum and Intraperitoneal Porcine C-Peptide

**Figure 2:**
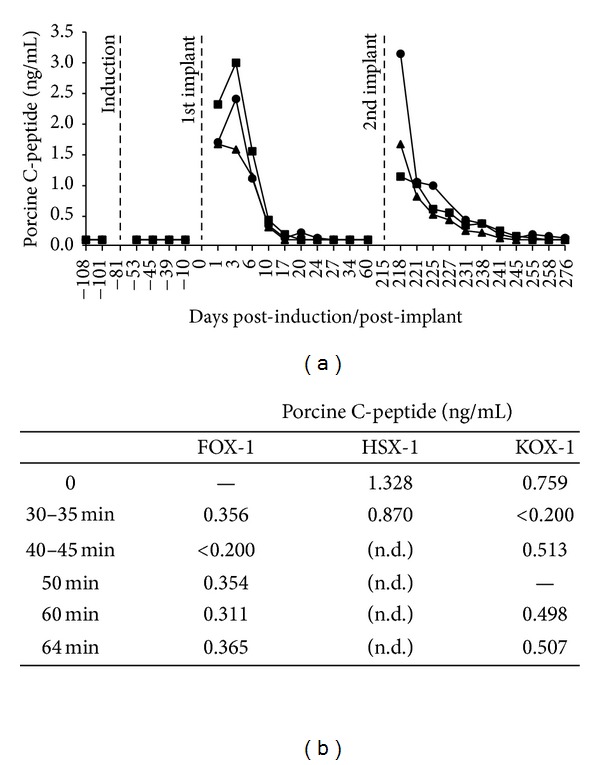
Serum porcine C-peptide levels of macrobead implanted dogs following 1st and 2nd implant [(a): FOX-1 (triangle); HSX-1 (square); KOX-1 (circle)]. Porcine C-peptide was also detected in the peritoneal fluid of implanted study animals following glucose/arginine challenge. (b) Porcine-C-peptide was not determined (n.d.) at all timepoints for HSX-1 because fluid was not collected beyond 30–35 minutes.

Random, nonfasting porcine C-peptide was not detected in the serum of any study animals before pancreatectomy or prior to macrobead implantation nor in the two control dogs throughout the study. Serum porcine C-peptide was, however, detected in the three macrobead implanted dogs after transplantation (Figure 2(a)). FOX-1 showed only minimal levels of C-peptide following both implant procedures with levels ranging from 0.1–0.2 ng/mL. C-peptide levels up to 0.31 ng/mL (HSX-1) and 0.29 ng/mL (KOX-1) were found 12-13 days following the first implant. On days 21-22 following 2nd implant, C-peptide levels of 0.34 ng/mL (HSX-1) and 0.45 ng/mL (KOX-1) were found. By days 82-83 post 2nd implant, only HSX-1 had any evidence of circulating porcine C-peptide (0.18 ng/mL).

Porcine C-peptide was detected in the peritoneal fluid of all three implanted dogs approximately 3 months prior to necropsy during an intraperitoneal glucose/arginine challenge procedure (Figure 2(b)). Serum C-peptide was not detected at this time in any of the three implanted dogs. Control animals did not undergo the peritoneal challenge or fluid collection procedure. At necropsy, porcine C-peptide was detected in the peritoneal fluid of HSX-1 and KOX-1 (0.41 ng/mL and 0.32 ng/mL, resp.) but not in the peritoneal fluid from FOX-1.

### 3.4. Body Weights

All animals lost body weight immediately after pancreatectomy. Over the next several months, study animals regained weight and surpassed preinduction levels. A slight increase in body weight was also observed in the immediate period following both macrobead implantations, as a result of macrobead weight and fluid therapy associated with the surgical procedures.

### 3.5. Necropsy

Complete necropsies were performed on all study animals on day 867 after macrobead implant. During the exploration of the peritoneal cavity it was noted that the majority of the macrobeads were free-floating and intact. Macrobeads were occasionally found to be either entangled in residual omentum tissue or contained within the pelvic omentum-like tissue (5–10% of macrobeads). Nonetheless, these entrapped macrobeads were free-floating within their tissue cavities. Few macrobeads from all three implanted dogs were also found to be lodged between the lobes of the liver. Only HSX-1 had any evidence of a broken macrobead(s): a few agarose macrobead fragments were observed embedded in a lobe of the liver. No broken macrobeads were observed in the other two implanted dogs.

**Figure 3:**
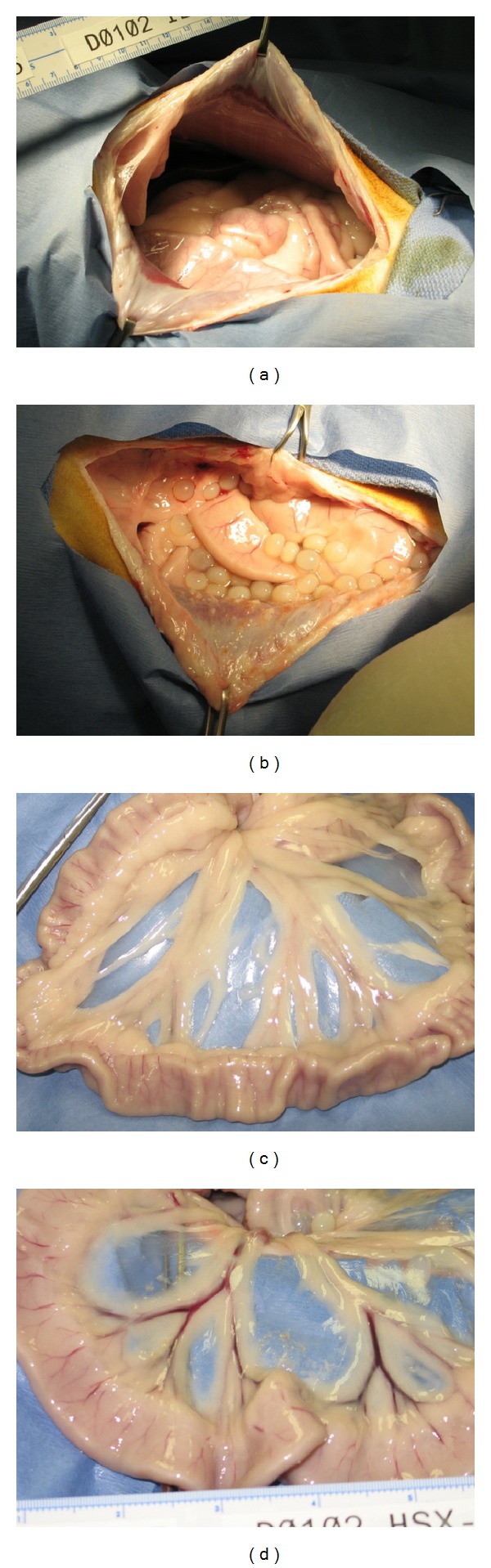
Macroscopic images taken at necropsy for a nonimplant control animal [ILX-1; (a), (c)] and a porcine islet macrobead recipient [HSX-1; (b), (d)]. (a)-(b) were taken at the initial opening. (c)-(d) are images of the mesentery of both animals.

Diffuse fibrotic plaques and mesothelial hyperplasia were common findings on the serosal surface of the abdominal wall and abdominal tissues. The proliferative lesions were limited to the serosal surfaces and did not appear to extend into organ parenchyma cells at sectioning. Macroscopic views of mesentery from implanted and control dogs were nearly identical (Figure 3) demonstrating the biocompatibility of the agarose macrobeads. Various lobes of the liver from all 3 implanted dogs presented with occasional indentations due to the presence of macrobeads.

### 3.6. Virology of Animal Samples and Tissues

During the surgical procedure at the time of second macrobead implant or sham surgery (control dogs), biopsies of tonsil and mesenteric lymph node and white blood cells were collected from all study animals for assessment of PERV.

At necropsy, nine tissues (bone marrow, C.N.S (brain), mesenteric lymph node, small intestine, heart, kidney, liver, lung, and PBMCs) and islet macrobeads were collected and screened by PCR for the presence of 6 different viruses. All tissues were negative for the presence of PCMV, PLHV, PERV, sHEV1 and sHEV2, and sEMCV. PERV specific sequences were detected in all islet macrobead samples. In samples from both control and implanted dogs, low level amplification signals for PCV were found in a number of different tissues suggestive of a cross-reaction with canine sequences.

### 3.7. Histopathology

Tissues and macrobeads collected at necropsy were processed and examined by a board-certified pathologist. Viable islet cells were not found in the macrobeads examined from the three implanted dogs. The majority of the macrobeads were found to contain amorphous basophilic, mineralized granules. Some macrobeads were surrounded by a proliferative inflammatory capsule characterized by a minimal to mild degree of fibrous collagen tissue. Peritoneal organs and tissues with a serosal surface had minimal to mild proliferative and inflammatory lesions. The lesions were composed of fibrous connective tissue covered with a thin layer of mesothelium or mesothelial cells. There were no systemic effects attributable to the presence of the macrobeads.

## 4. Discussion

The results of this study demonstrate the safety of transplanting agarose-agarose porcine islet macrobeads in a fully discordant large animal model as a treatment for insulin dependent diabetes. Importantly, specific immunosuppressive therapy was not administered, although pravastatin was given for its mild anti-inflammatory properties. All three dogs that received porcine islet macrobeads were insulin-free for approximately 2 weeks following the first implant procedure. Preimplant insulin requirements were reached over the following week such that by three weeks after implantation all animals were again on full insulin requirements. Remarkably, the implanted dogs did not reach pre-insulin requirements following the second implant, until significantly longer periods of time. In fact, one animal still had not reached preimplant insulin requirements, at necropsy, on day 652 following 2nd implant.

A significant issue with any xenotransplantation procedure is the possibility of xenozoonosis. In the case of porcine islets, porcine endogenous retrovirus (PERV) is encoded throughout the porcine genome, and because it is unlikely to be removed, this virus has generated considerable attention of the xenotransplantation community and regulatory authorities (see review by Denner and Tonjes [16]). Although PERV was shown to infect human cells* in vitro* [17], there is no evidence of transmission to humans including farmers, veterinarians, or abattoir workers, all of whom are exposed daily to numerous porcine secretions, excretions, blood, and tissues. More than 80 years of using porcine insulin in the clinic has also not revealed any transmission of PERV. Preclinical studies in nonhuman primates have also not detected any evidence of PERV transmission [18] even with high dose immunosuppression and high titers of PERV [19]. Also assuring are the negative human clinical findings of PERV transmission to date including burn patients treated with porcine skin [20], patients with hemophilia treated with porcine factor VIII [21], and patients receiving extracorporeal porcine hepatocyte therapy [22–24], as well as from two clinical trials of porcine islets following implantation [25, 26]. Nonetheless, the accidental transmission of animal viruses and PERV in particular mandate a carefully thought out strategy to manage such risks [27, 28].

The ability of the islet macrobeads to survive extended culture periods up to at least one year [6] provides ample opportunity to not only determine the insulin production from each batch of macrobeads but to also screen the macrobeads for evidence of microbiological and viral pathogens. We have recently demonstrated the proliferation of macrobead encapsulated porcine insulin-producing cells over the first few weeks of culture [29]. During this time, insulin production increases from starting levels and gradually stabilizes. This allows a determination of the precise insulin-producing capacity of encapsulated islets and the subsequent implantation of a patient-tailored dose of macrobeads to meet individual insulin demands.

As noted above, the islet macrobeads are also routinely screened for numerous pathogens prior to implantation into study animals. Traditional organ allografting techniques do not allow for the extended testing times required to assure the absence of xenorelated infectious diseases, and until now, the promise of islet xenotransplantation has been largely unrealized by this restraint. The agarose-agarose islet macrobead affords ample time for extensive microbiological safety testing. We consider this an advanced technique to the antemortem screening of donor animals because the tissue to be transplanted can be tested directly. This is a significant technological advance and one with the potential to finally make xenotransplantation a reality. Furthermore, additional patient safety is obtained by not requiring the immunosuppression of macrobead recipients [30].

Similar to the findings discussed above, we have not found any evidence of viral transmission, including PERV, in diabetic rats implanted with porcine islet macrobeads [6]. In the current study, viral transmission was also not found in biopsies of mesenteric lymph node tissue and tonsil tissue and peripheral blood mononuclear cells on day 215 after the 1st implant (immediately prior to implanting the second dose of macrobeads).

Additionally, numerous tissues were collected at necropsy from all study dogs. At this time, islet macrobead implanted dogs had been exposed to porcine tissue for 2.4 years. All tissues were found to be negative for the screened viral sequences although some low level amplification was observed for PCV in some tissues of all dogs including the nonimplanted control animals. As low level amplification signals for the PCV target were detected in both negative control and macrobead implanted animals, it is likely that this finding is due to a cross-reaction with canine specific sequences. The negative findings from the extensive viral screening of the islet macrobeads prior to implantation also support the notion that porcine islet macrobead implantation, under the conditions of this study, can be performed safely and does not result in trans-species viral transmission.

Consistent with a previous study [31], the islet macrobeads were found to be extremely biocompatible. At necropsy, most islet macrobeads were intact and free-floating in the peritoneal cavity. Only one animal had any evidence of a broken macrobead(s). These results demonstrate the ability of the islet macrobeads to resist breakage* in vivo*. Perhaps the most impressive finding at necropsy was the mild degree of inflammatory reaction in the peritoneal cavity which was not thought to impair organ function. Furthermore, these data also demonstrate the capacity of the abdomen to accommodate a large number of macrobeads. Ongoing improvements in the insulin secretion capacity of each islet macrobead continue to allow for the implantation of fewer macrobeads in order to replace exogenous insulin. Finally, current clinical trials of a similarly sized mouse renal adenocarcinoma-containing agarose macrobead for the inhibition of various cancers have shown the human abdomen to easily house several thousand such macrobeads [32, 33].

These data support the feasibility of transplanting agarose-agarose porcine islet macrobeads as a treatment strategy for insulin dependent diabetes mellitus. The macrobeads are resistant to breakage and degradation and are nontoxic and biocompatible. The ability to incubate the porcine islet macrobeads for extended periods in order to prescreen the islets for numerous pathogens further advances the safety profile of this therapy. Critically, no evidence of viral transmission was observed after long-term implantation in diabetic study animals.

## Figures and Tables

**Table 1:** Preimplant microbiological and vial screening of porcine islet macrobeads.

Test description	Testing facility	Methodology
Total product bioburden testing	Microtest Laboratories, Inc.	ANSI/AAMI/ISO 11737-1 USP 〈61〉 membrane filtration
*Salmonella sp.* screening	Microtest Laboratories, Inc.	USP 〈62〉
Sterility	Microtest Laboratories, Inc.	USP 〈71〉 membrane filtration
Mycoplasma	Microtest Laboratories, Inc.	Direct cultivation methods Cell culture methods
Bacterial endotoxin	Microtest Laboratories, Inc.	Kinetic chromogenic
Porcine reproductive and respiratory syndrome virus (PRRSV)	VDL	RT-PCR
Swine influenza virus (SIV)	VDL	RT-PCR
Porcine endogenous retrovirus (PERV)	VDL	RT-PCR
Porcine enterovirus (PEV)	VDL	RT-PCR
Porcine respiratory corona virus (PRCV)	VDL	RT-PCR (Qiagen 283615)
Transmissible gastroenteritis virus (TGEV)	VDL	RT-PCR (Qiagen 283615)
Porcine circovirus types 1 and 2 (PCV)	VDL	Multiplex PCR
Porcine lymphotropic herpes virus type-1 (PLHV-1)	VDL	PCR
Swine hemagglutinating encephalomyelitis virus (sHEV)	VDL	RT-PCR
Porcine parvovirus (PPV)	VDL	PCR
Porcine cytomegalovirus (PCMV)	VDL	PCR
*Mycoplasma hyopneumoniae *	VDL	PCR
Pseudorabies virus (PRV)	VDL	Virus isolation
Encephalomyocarditis virus (EMCV)	VDL	Virus isolation
Rotavirus (type A)	VDL	Antigen capture ELISA
*Chlamydia* spp.	VDL	Antigen capture ELISA

**Table 2:** Microbiological screening performed at necropsy on retrieved islet macrobeads and study animal samples and tissues.

Test description	Testing facility	Methodology
Sterility	Microtest Laboratories, Inc.	USP 〈71〉 membrane filtration
Mycoplasma	Microtest Laboratories, Inc.	Direct cultivation methods Cell culture methods
Bacterial endotoxin	Microtest Laboratories, Inc.	Kinetic chromogenic
Porcine endogenous retrovirus (PERV)	BioReliance	qRT-PCR
Porcine circovirus types 1 and 2 (PCV)	BioReliance	qRT-PCR
Porcine lymphotropic herpes virus type-1 (PLHV-1)	BioReliance	qRT-PCR
Swine hepatitis E virus (sHepE)	BioReliance	qRT-PCR
Porcine cytomegalovirus (PCMV)	BioReliance	qRT-PCR
Swine encephalomyocarditis virus (sEMCV)	BioReliance	qRT-PCR

**Table 3:** Individual dog insulin requirements and islet macrobead information at first and second implant.

				Porcine islet macrobead information			
	Animal ID	Body weight (kg)	Daily insulin requirement (IU)	Daily insulin production (IU/bead)	Number of macrobeads implanted	Total daily insulin (IU) from implanted macrobeads	Weight of implanted macrobeads (kg)
First implant	FOX-1	12	14	0.046	1209	55.6	0.32
	HSX-1	11	13	0.046	1118	51.4	0.31
	KOX-1	12	16	0.046	1380	63.5	0.36

Second implant	FOX-1	13	15	0.042	1096	46.0	0.29
	HSX-1	14	26	0.042	1904	79.9	0.49
	KOX-1	14	16	0.042	1170	49.1	0.30
